# DNA-dependent targeting of cell nuclei by a lupus autoantibody

**DOI:** 10.1038/srep12022

**Published:** 2015-07-09

**Authors:** Richard H. Weisbart, Grace Chan, Gwen Jordaan, Philip W. Noble, Yanfeng Liu, Peter M. Glazer, Robert N. Nishimura, James E. Hansen

**Affiliations:** 1 Department of Research, Veterans Affairs Greater Los Angeles Healthcare System, Sepulveda, CA 91343; 2 Department of Therapeutic Radiology, Yale School of Medicine, New Haven, CT 06520; 3 Department of Genetics, Yale School of Medicine, New Haven, CT 06520; 4 Yale Cancer Center, Yale School of Medicine, New Haven, CT 06520; 5 Department of Neurology, University of California, Los Angeles, CA 90095

## Abstract

A nuclear-penetrating lupus anti-DNA autoantibody, 3E10, has been found to inhibit DNA repair and selectively kill certain cancer cells that are highly vulnerable to DNA damage. In addition, a 3E10 single chain variable fragment (scFv) has been developed for use as a delivery vehicle to carry therapeutic cargo proteins into cell nuclei. A greater understanding of the mechanism by which 3E10 penetrates cell nuclei is needed to help determine the scope of its potential therapeutic applications. Here we show that the presence of extracellular DNA significantly enhances the nuclear uptake of 3E10 scFv. In addition, we find that 3E10 scFv preferentially localizes into tumor cell nuclei *in vivo*, likely due to increased DNA in the local environment released from ischemic and necrotic regions of tumor. These data provide insight into the mechanism of nuclear penetration by 3E10 and demonstrate the potential for use of 3E10 in therapeutic approaches to diseases ranging from malignancy to ischemic conditions such as stroke.

Antibody-based immunotherapy has improved treatment outcomes in diseases ranging from rheumatologic conditions to cancer, and efforts are underway to optimize these approaches to yield even greater clinical impact. Most immunoglobulins are unable to penetrate into living cells and therefore cannot be used to target key intracellular antigens or pathways, and this has been a critical factor that has limited the expansion of the use of antibodies in molecular therapy. However, a select group of lupus autoantibodies penetrate into living cells^1^ and have a multitude of potential applications in molecular therapy.

An unusual lupus anti-DNA antibody that penetrates cell nuclei without causing any apparent harm to normal cells or tissues, 3E10, has been found to inhibit DNA repair and thereby selectively kill cancer cells that are vulnerable to DNA damage due to BRCA2-deficiency^2^. In addition, an optimized 3E10 single chain variable fragment (scFv) with an enhancing mutation that improves its efficiency of nuclear penetration has been developed as a molecular delivery vehicle and has carried cargo proteins such as p53 and Hsp70 into cell nuclei *in vitro* and *in vivo*^3^,^4^,^5^,^6^,^7^. The development and optimization of cell-penetrating antibody technology could potentially transform antibody-based therapy, but in order to better delineate the scope of the possible therapeutic applications of 3E10 scFv a greater understanding of the mechanism by which it penetrates cell nuclei is needed.

Some cells take up immunoglobulins and immune complexes through Fc-receptor mediated endocytosis^8^,^9^, but any major role of endocytosis in the mechanism of cellular penetration by 3E10 scFv has been ruled out. 3E10 scFv penetrates living cells in a highly efficient manner despite not having an Fc region, which indicates Fc receptors are not required for cellular uptake of 3E10 scFv^10^,^11^. Moreover we have observed that methyl-β-cyclodextrin, a chemical inhibitor of endocytosis, does not perturb nuclear penetration by 3E10 scFv (Hansen, J. E., Nishimura, R. N. & Weisbart, R. H. Unpublished observation). Taken together these findings suggest that 3E10 scFv penetrates cells through a method that is independent of endocytosis.

The ability of 3E10 to bind DNA is critically linked to its ability to penetrate cell nuclei. 3E10 is a cationic antibody, like most anti-DNA antibodies, and this contributes to its ability to bind to negatively charged DNA. For example, CDR3 of the 3E10 V_H_ contains the sequence RGLLLDY, and mutation of the arginine to an aspartic acid residue takes away a positive charge and abrogates DNA binding by 3E10^10^. By contrast, removing a negative charge from CDR1 of the 3E10 V_H_ by mutating an aspartic acid residue to asparagine greatly enhances the affinity of 3E10 for DNA^11^. Remarkably, these mutations in 3E10 that interfere with its DNA binding activity also impair its transduction into cell nuclei, and similarly the mutations that increase its DNA binding affinity result in improved nuclear transduction efficiency^10^. The recognition of the association between DNA binding and nuclear penetration by 3E10 led to an analysis of the possible contributions of nucleoside salvage pathways to nuclear uptake of 3E10, and nuclear penetration by 3E10 scFv was found to require the presence of a specific nucleoside transporter, equilibrative nucleoside transporter-2 (ENT2), in cells^11^.

Overall, the previous work strongly suggests that 3E10 scFv penetrates cell nuclei by binding to DNA or its components and then following it into cells through the ENT2 nucleoside salvage pathway. If this hypothesis is correct then 3E10 scFv should not be able to penetrate into cells in the absence of extracellular DNA. Moreover, addition of extracellular DNA would be expected to enhance nuclear uptake of 3E10 scFv. We therefore set out to test our hypothesized model of nuclear penetration by 3E10 scFv by evaluating the impact of the presence of extracellular DNA on efficiency of nuclear uptake of 3E10 scFv as described below.

## Results

### 3E10 scFv does not penetrate efficiently into cell nuclei in the absence of extracellular DNA

**Figure 1:**
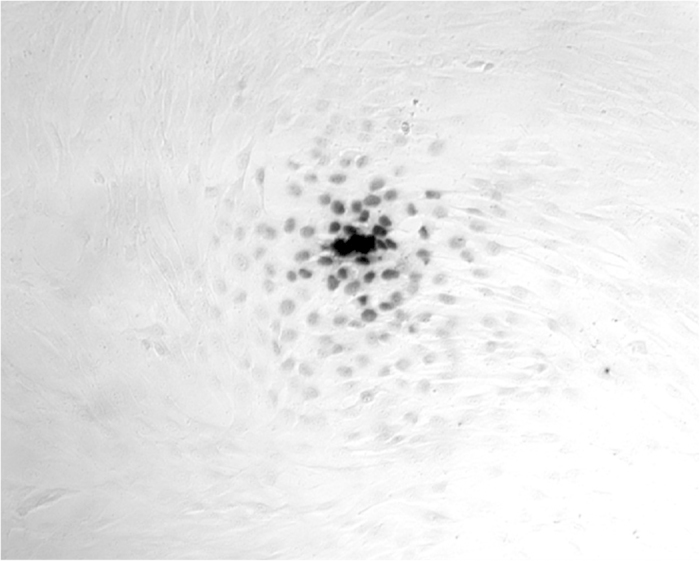
3E10 scFv penetrates most efficiently into living cells surrounding a dead cell. GM02605 fibroblasts were washed with serum free media and then treated with 10 μM 3E10 scFv for one hour, followed by anti-Myc immunostaining to detect nuclear penetration by 3E10 scFv. Nuclear penetration by 3E10 scFv was restricted to cells in close proximity to what morphologically appears to be a dead cell, suggesting that a factor released by dead cells promotes nuclear uptake of the fragment.

We sought to test the efficiency of nuclear penetration by 3E10 scFv into cells in the absence of extracellular DNA, and for these studies selected the GM02605 human fibroblast cell line because it maintains a high degree of viability (>99%) with minimal cell death even while maintained in culture for several days. With minimal cell death in culture the confounding effects of DNA released by dead cells are minimized. The GM02605 cells were washed with serum free media and then treated with 10 μM 3E10 scFv for one hour, after which cells were fixed and immunostained for presence of the fragment. Remarkably, 3E10 scFv did not penetrate into most cells. Instead, 3E10 scFv was found only in the nuclei of cells centered around what morphologically appeared to be rare dead cells, with a gradient effect observed with decreased amounts of intranuclear fragment detected with increasing distance from the central dead cell. A representative image demonstrating this effect is shown in Fig. 1. This observation was consistent with a factor released locally by dead cells enhancing nuclear penetration by 3E10 scFv into surrounding cells.

### Addition of cell lysate promotes nuclear uptake of 3E10 scFv

**Figure 2:**
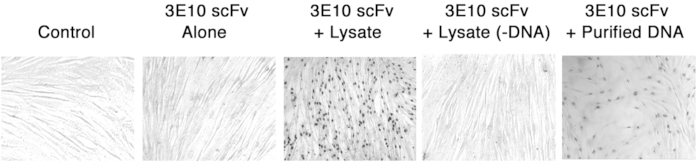
Extracellular DNA facilitates penetration of 3E10 scFv into cell nuclei. GM02605 fibroblasts were washed with serum free media and then treated with control buffer alone or 10 μM 3E10 scFv in the presence of control buffer, cell lysate, DNA-depleted cell lysate, or purified DNA for one hour, followed by anti-Myc immunostaining to detect nuclear penetration by 3E10 scFv. Nuclear penetration into ~100% of the cells was only observed in the presence of cell lysate or purified DNA.

To test the hypothesis that a factor released by dead cells enhances nuclear uptake of 3E10 scFv we compared the efficiency of nuclear penetration of the fragment into the GM02605 fibroblasts in the presence or absence of a cell lysate. As shown in Fig. 2, in the absence of the cell lysate minimal nuclear uptake of 3E10 scFv was observed. However, the addition of cell lysate facilitated nuclear penetration by 3E10 scFv into ~100% of the cells. These results further support the hypothesis that a factor released by dead cells and contained in cell lysate promotes nuclear uptake of 3E10 scFv.

### DNA-depleted cell lysate does not enhance nuclear uptake of 3E10 scFv

We hypothesized that DNA is the critical factor in cell lysate that promotes nuclear uptake of 3E10 scFv. To test this, the GM02605 fibroblasts were treated with 10 μM 3E10 scFv in the presence of cell lysate that had been passed through a Centricon cellulose filter with a molecular weight cut off of 10,000 kDa to remove DNA content. In contrast to the complete cell lysate, the DNA-depleted lysate did not enhance nuclear uptake of 3E10 scFv (Fig. 2), which strongly supports the hypothesis that DNA is the relevant factor contributing to nuclear penetration by the fragment.

### Addition of purified DNA promotes nuclear uptake of 3E10 scFv

To confirm that extracellular DNA enhances nuclear penetration by 3E10 scFv, we next treated the GM02605 fibroblasts with 10 μM 3E10 scFv in the presence of purified DNA (0.5 g/L). As shown in Fig. 2, addition of purified DNA to the media significantly enhanced the efficiency of penetration by 3E10 scFv into cell nuclei. Taken together, these data indicate that nuclear penetration by 3E10 scFv is enhanced by the presence of extracellular DNA.

### 3E10 scFv preferentially localizes into tumor cells *in vivo*

**Figure 3:**
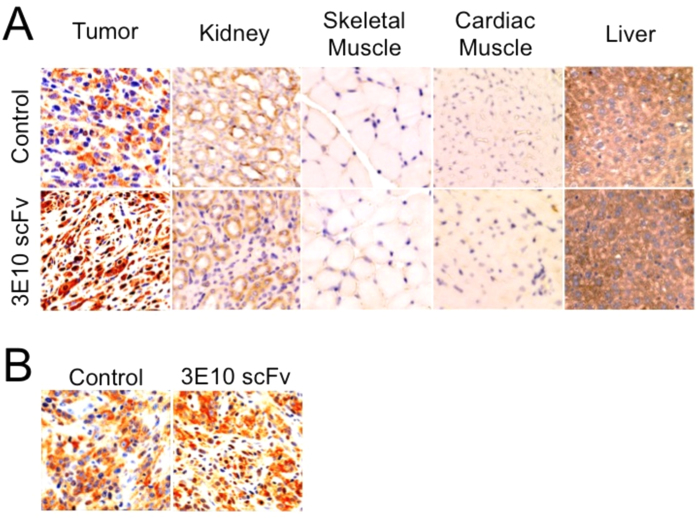
3E10 scFv localizes to tumor cell nuclei *in vivo*. Immunodeficient mice bearing subcutaneous U87 human glioma xenografts were treated by intraperitoneal injection of control buffer or 3E10 scFv. Mice were sacrificed 4 or 24 hours after treatment, and tumors and select normal tissues were immunostained for the presence of 3E10 scFv. Brown nuclear stain indicates presence of 3E10 scFv, while blue nuclear stain is negative. (**A**) Four hours after treatment 3E10 scFv was detected in the nuclei of the U87 tumor cells but was not detected in tissues of major organs including heart, kidney, skeletal muscle, and liver. These results are consistent with preferential uptake of 3E10 scFv into tumors. (**B**) Twenty-four hours after treatment 3E10 scFv was still detectable in the tumors, demonstrating the stability of the uptake into tumor nuclei.

As described above, 3E10 scFv has potential applications in cancer therapy both as a delivery vehicle and as an inhibitor of DNA repair. After confirming that 3E10 scFv penetrates cell nuclei most efficiently in the presence of extracellular DNA, we hypothesized that the fragment would preferentially localize into the nuclei of tumor cells *in vivo* due to an expected higher concentration of extracellular DNA in the tumor vicinity released from dead cells in regions of tumor ischemia and necrosis. To test this, subcutaneous U87 human glioma xenografts were generated in immunodeficient mice, and once tumors grew to size of ~100 mm^3^ mice were treated with intraperitoneal injection of control buffer or 3E10 scFv. Mice were then sacrificed 4 or 24 hours after treatment, and tumors and select normal tissues were immunostained for the presence of 3E10 scFv. Four hours after treatment 3E10 scFv was not detected in the cell nuclei of normal tissues including heart, kidney, skeletal muscle, and liver. By contrast, cell nuclei in the tumor xenografts stained positive for presence of 3E10 scFv (Fig. 3A). 3E10 scFv was also detected in the tumors 24 hours after treatment, demonstrating the stability of the uptake into tumor nuclei (Fig. 3B). These results are consistent with preferential uptake of 3E10 scFv into tumors.

## Discussion

The lupus anti-DNA autoantibody 3E10 has the unusual ability to penetrate into the nuclei of living cells, and a 3E10 single chain variable fragment, 3E10 scFv, has been developed for use in molecular therapy^2^,^3^,^4^,^5^,^6^,^7^. Elucidation of the mechanism by which 3E10 scFv penetrates cell nuclei is important to determining the full scope of its potential therapeutic applications. Our previous work with 3E10 scFv established that its mechanism of nuclear uptake is independent of endocytosis pathways^10^,^11^ (Hansen, J. E., Nishimura, R. N. & Weisbart, R. H. Unpublished observation). Instead, we have found that nuclear penetration by 3E10 scFv is critically dependent on its ability to bind DNA, and that 3E10 scFv is unable to penetrate effectively into cells that lack the ENT2 nucleoside transporter that is present in both outer plasma and nuclear membranes^11^,^12^. In the present manuscript we have now shown that extracellular DNA is required for penetration of 3E10 scFv into live cell nuclei. Based on the combination of findings described above, we believe it is most likely that 3E10 scFv penetrates cell nuclei by first binding to extracellular DNA or its degradation products and then following them into cell nuclei through the ENT2 nucleoside salvage pathway. This is an unusual pathway of nuclear protein transuction and additional studies are required to further elucidate the details of this mechanism, but all of the data generated to date are consistent with this hypothesis.

Another important finding that we report in the present study is that 3E10 scFv preferentially localizes to tumor xenografts *in vivo*. To the best of our knowledge the results presented here are the first to compare the distribution of 3E10 scFv in malignant and normal tissue *in vivo*. The tissue distribution of 3E10 scFv in FVB mice without tumors has been previously evaluated, and consistent with our current results minimal penetration of the 3E10 scFv into normal tissues was observed in FVB mice treated with tail vein injection of the fragment. Specifically, 3E10 scFv did not localize into brain, lung, intestine, spleen, liver, pancreas, ovary, or skin, and was found in only a small percentage of skeletal muscle cell nuclei and in rare renal tubule cells^13^. In a separate study, a 3E10 scFv-Hsp70 (Fv-Hsp70) fusion protein was administered intravenously to rats after induction of a middle cerebral artery stroke in order to test the hypothesis that 3E10 scFv would be able to deliver a cytoprotective dose of Hsp70 to regions of ischemic injury. Remarkably, the Fv-Hsp70 fusion protein selectively penetrated into ischemic brain, reduced the infarct volumes, and improved neurologic outcomes^6^. When taken together, these previous observations coupled with our present findings strongly support the hypothesis that 3E10 scFv selectively localizes to ischemic or injured tissues such as portions of skeletal muscle subject to contractile injury, brain in the distribution of a stroke, or ischemic and partially necrotic tumors. In the context of our present finding that extracellular DNA is required for nuclear penetration by 3E10 scFv, we believe that the preferential targeting of 3E10 scFv to damaged or ischemic tissues is explained by the increased concentration of extracellular DNA released by dying cells in these regions.

The selective localization of 3E10 scFv into damaged or ischemic tissues may explain in part the remarkable lack of systemic toxicity associated with 3E10 or 3E10 scFv-fusion proteins in previous *in vivo* studies^2^,^3^,^6^,^7^,^13^, and this finding further establishes the potential to use 3E10 scFv in clinical applications wherein delivery of therapeutic agents to injured or ischemic tissues is needed. Moreover, the recognition of the enhancing effect of extracellular DNA on nuclear penetration by 3E10 scFv allows one to consider strategies to further optimize uptake of 3E10 scFv and its fusion proteins into target tissues. For example, co-administration of 3E10 scFv with a targeted dose of radiation to tumor may yield even greater tumor uptake by the fragment due to increased release of DNA by tumor cells dying after exposure to the radiation. Overall, the data presented herein provide additional evidence of the association between cellular uptake of DNA and nuclear penetration by 3E10 and further demonstrate the potential for use of 3E10 scFv in therapeutic approaches to diseases ranging from malignancy to ischemic conditions such as stroke.

## Methods

### Production and purification of 3E10 scFv

3E10 scFv was produced in *P. pastoris* and purified as previously described^2^.

### Cell lines

The GM02605 human fibroblast cell line (Coriell Biorepository, Camden, NJ) grows to confluence in 96-well tissue culture plates with remarkably high viability (>99% viability maintained over several days of growth as determined by propidium iodide exclusion assay). Cells were grown in MEM with 15% FCS and washed with MEM without serum before incubation with 10 μM 3E10 scFv for one hour. Nuclear penetration by 3E10 scFv was then examined by anti-Myc immunostaining as previously described^11^.

### Cell lysate

COS-7 cell lysate was prepared by subjecting cells to multiple freeze-thaw cycles in liquid nitrogen. Cell debris was removed by centrifugation. DNA-depleted COS-7 cell lysate was prepared by passing the lysate through a Centricon cellulose filter with a molecular weight cut off of 10,000 kDa.

### DNA

Purified calf thymus DNA sheared to an average length of 2000 bp was purchased from Invitrogen (Ultrapure, Invitrogen, Carlsbad, CA).

### Human glioma xenografts

U87 human glioma subcutaneous xenografts were generated in nude mice as previously described^2^. When tumors reached size of ~100 mm^3^ mice were treated with intraperitoneal injection of control PBS buffer or 0.8 mg 3E10 scFv in PBS. Mice were sacrificed 4 or 24 hours after treatment, and tumors and selected normal tissues were fixed in formalin and embedded in paraffin. Tissues were then surveyed for nuclear penetration by 3E10 scFv by immunohistochemistry (IHC). Tissue sections were deparaffinized, rehydrated, and incubated at 95–99 C for 30 minutes for epitope retrieval. Sections were washed, blocked with peroxidase, and probed with a 9E10 anti-Myc (abcam, Cambridge, UK) primary antibody directed at the C-terminal Myc tag in 3E10 scFv followed by additional washes and then incubation with a labeled polymer-HRP secondary antibody (Envision, Dako, Carpenteria, CA). After additional washes color development was performed using DAB followed by counterstaining with hematoxylin. All *in vivo* studies were conducted in accordance with institutional guidelines. The protocol for the *in vivo* work was approved by Yale University’s Institutional Animal Care and Use Committee.

## Additional Information

**How to cite this article**: Weisbart, R. H. *et al.* DNA-dependent targeting of cell nuclei by a lupus autoantibody. *Sci. Rep.* **5**, 12022; doi: 10.1038/srep12022 (2015).
